# The Psychological Impact of Isolation on Hospitalised Patients with COVID-19 Infection in the UAE

**DOI:** 10.1007/s44197-022-00070-4

**Published:** 2022-10-15

**Authors:** Nahida Nayaz Ahmed, Nirmin F. Juber, Reem AlKaabi, Fatema AlShehhi, Mohamed AlObeidli, Ahlam Salem, Alaa Galadari, Shamil Wanigaratne, Amar Ahmad

**Affiliations:** 1 Department of Psychiatry, Abu Dhabi Health Services Company, Abu Dhabi, United Arab Emirates; 2 Public Health Research Centre, New York University Abu Dhabi, P.O. Box 129188, Abu Dhabi, United Arab Emirates; 3 Departments of Psychology, Zayed University, Abu Dhabi, United Arab Emirates; 4 Research Section, National Rehabilitation Centre, Abu Dhabi, United Arab Emirates

**Keywords:** COVID-19, Mental health, Depression, Anxiety, Psychological distress, UAE

## Abstract

Infection prevention and control measures for COVID-19 may include immediate admission to an isolation facility for the infected. However, the mental health impact of this isolation worldwide is not fully documented. This study aims to contribute to global data on the psychological impact of COVID-19 and to be the first study to assess psychological distress among hospitalised patients with COVID-19 in the UAE. Using a cross-sectional study design on 132 hospitalised patients, we found that 90% of participants scored within the normal levels for psychological distress. The length of stay was associated with higher levels of psychological distress and those aged 41–60 years had lower levels of psychological distress compared to the 31–40 years group. Our results contributed to global data on the psychological impact of COVID-19 and may help to identify those at risk for psychological distress due to COVID-19 hospitalisation for targeted prevention and future pandemic preparedness plans.

## Introduction

The Coronavirus disease of 2019 (COVID-19) outbreak started in December 2019 in Wuhan, China, and swept through the globe rapidly, achieving WHO Pandemic status by mid-March 2020 [1]. COVID-19-related restrictions (infection prevention measures) have been applied worldwide and are regarded as an important measure to tackle the COVID-19 spread. By April 2020, more than a third of the global population was under COVID-19 movement restrictions or lockdowns [2]. COVID-19 restrictions, such as medical isolation, travel restrictions, and outdoor activity limitations have. In many cases, led to elevated levels of stress and anxiety among those impacted [3].

The link between viral infection and psychological effects among the survivors has been established [4]. Neuropsychiatric links have been established between Severe Acute Respiratory Syndrome (SARS) in the early phase of the illness and depression, anxiety, panic disorder, suicidality, as well as delirium, and psychosis [5]. Data on the mental health impact on the community at large is emerging from many countries, and the prevalence of anxiety and depression has been reported to be more than doubled compared to pre-COVID-19 periods as reported in Mexico, the United Kingdom, and the United States [6]. A survey by the Indian Psychiatric Society reported a 20% increase in mental illness in India since the pandemic [7]. Therefore, researchers have urged that countries learn from the pandemic and recognize the importance of public mental health and integrate it into public health preparedness and disaster planning [8].

The United Arab Emirates (UAE) has been successful in limiting the spread of COVID-19 through several preventive measures, including immediate admission to an isolation facility for those infected with Coronavirus [9]. Isolation among hospitalised patients with COVID-19 is needed due to their high level of transmissibility [10]. Being isolated in the hospital may produce psychological instability among those with COVID-19 infection, due to a lack of interactions with families and friends [11], as well as due to the COVID pandemic situation [12]. A previous study showed that during the isolation period at the hospital, 57.2 and 52.2% of hospitalised patients with COVID-19 infection had anxiety and depressive symptoms, respectively [10]. A cross-sectional study in an Iranian community also revealed that compared to the community samples, hospitalised patients with COVID-19 infection had significantly higher anxiety, depression, and stress levels as measured using The Depression, Anxiety and Stress Scale—21 Items (DASS-21) [13].

Studies from various countries on the mental health impact of COVID-19 are being published, painting a global picture. In the Middle East region, studies on the psychological impact of the COVID-19 pandemic among the general population have been published [14, 15], however, the study on the psychological impact of hospitalised patients with COVID-19 infection in this region is very limited. Therefore, this study aims to contribute to global data on the psychological impact of COVID-19 and to be the first study to assess the levels of depression, anxiety, and stress among hospitalised patients with COVID-19 infection in the UAE.

## Methods

### Study Design, Study Population, and Participants

We conducted a cross-sectional study via a researcher-administered questionnaire through a telephone call to all consenting and medically stable COVID-19 patients hospitalised in isolation units in three health care facilities in Abu Dhabi City, UAE. In this study, we used a purposive or subjective sampling methods, a type of non-probability convenience sampling technique in which the sample is selected due to their characteristics and the objective of the study [16]. We studied 132 patients who were diagnosed with COVID-19 positive by reverse transcription polymerase chain reaction (RT-PCR) laboratory test and were admitted to COVID-19 isolation units in three participating health care facilities in Abu Dhabi city, UAE. We included all patients aged 18 years or above, clinically stable patients or vitals within normal limits, can communicate without difficulty, cognitively able to comprehend and respond to the questionnaire. Our study excluded those less than 18 years of age, clinically unstable patients (on ventilators or hemodynamic instability etc.), and cognitively impaired patients.

### Data Collection

Recruitment of participants was carried out by approaching leads of treatment teams in charge of the patients admitted to COVID-19 isolation wards at the enlisted health care facilities from July 2020 to December 2020. Consenting teams disseminated a copy of the consent form to the patients under their care. The consent form was available in English/Urdu/Hindi and Arabic. Patients who consent to participate received a telephone call during their hospital stay from the research team, who administered the questionnaire to the patients and recorded the responses on an electronic portal managed by the research team. Patient medical records were not accessed, nor the patient’s identifiers were recorded for the purpose of this study. A coordinator and co-researchers were available to answer questions concerning the survey by email or phone. The study was approved by the Department of Health, Abu Dhabi Research Ethics Committee.

### Questionnaire and Psychological Distress Scales

The survey instrument comprised of questions on demographic characteristics of post-traumatic stress, measured by the Impact of Event Scale-Revised (IES-R), a 22-item self-assessment questionnaire assessing the severity of distress because of a traumatic event [17]. Depression, anxiety, and stress levels measured by DASS-2 [18], a 21-item self-assessment questionnaire. These two instruments have been used worldwide to measure the impact of COVID-19 pandemic on mental health [19]. For each element in DASS-21 scale, the participant indicated how much he/she was disturbed or distressed in the last week by each of the difficulties listed using a Likert scale ranging from 0 "Not at all" to 4 "Extremely”. A total score was calculated by adding the scores of all elements (range 0–88). The total depression, anxiety, and stress scores using IES-R scale were reported in continuous form, while the score of each element using DASS-21 scale was categorized into five categories; normal, mild, moderate, severe, and extremely severe. Due to significant correlations between IES-R and DASS-21 scales in capturing psychological distress, we assessed the levels of psychological distress measured by the IES-R scale as an outcome in our regression analysis. Furthermore, to ensure its reliability, we evaluated the Cronbach’s alpha score of the IES-R in this study.

## Statistical Analysis

The sociodemographic distribution was calculated in terms of contingency tables of the *n* (%). The Mann–Whitney *U* test or Kruskal–Wallis test was performed to examine variation in post-traumatic stress disorder levels according to sociodemographic category. Spearman's correlation with 95% confidence interval (95% CI) was performed to analyse the association between the level of psychological distress measured by IES-R scale and the levels of depression, anxiety, and stress measured by DASS-21 scale.

Univariate and multivariate ordinal logistic (proportional odds) regression models were fitted with the level of psychological distress as an outcome. The predictors were age group, gender, nationality, marital status, presence of medical condition or comorbidity, and length of hospital stay. Odds ratios (OR) and 95% confidence interval (95% CI) were reported with the corresponding Wald’s *z* and *p* values. Statistical analyses were performed in R version 4.0.5 [20]. All applied statistical tests were two-sided; *p* value < 0.05 were considered statistically significant. No adjustment for multiple comparisons was made.

## Results

Table 1 shows DASS-21, frequency (percentage) of the level of stress, depression, and anxiety of the COVID-19 patients included in this statistical analysis. 93.2, 80.3, and 95.5% of the patients were in a normal range of depression, anxiety, and stress, respectively. Table 1 illustrates the cut-off values used to diagnose the level of expression, anxiety, and stress.

**Table 1:** DASS-21 frequency (percentage) of the level of stress, depression, and anxiety among study participants, and their DASS-21 cut off scores

	Depression	Anxiety	Stress
DASS-21 frequency (percentage) of the level of Stress, Depression, and anxiety			
Normal	123 (93.2)	106 (80.3)	126 (95.5)
Mild	5 (3.8)	12 (9.1)	5 (3.8)
Moderate	4 (3)	10 (7.6)	1 (0.8)
Severe	0 (0)	4 (3)	0 (0)
Extremely severe	0 (0)	0 (0)	0 (0)
DASS-21 cut off scores			
Normal	0–9	0–7	1–4
Mild	10–13	8–9	15–18
Moderate	14–20	10–14	19–25
Severe	21–27	15–19	26–33
Extremely severe	28 +	20 +	34 +

**Fig. 1:**
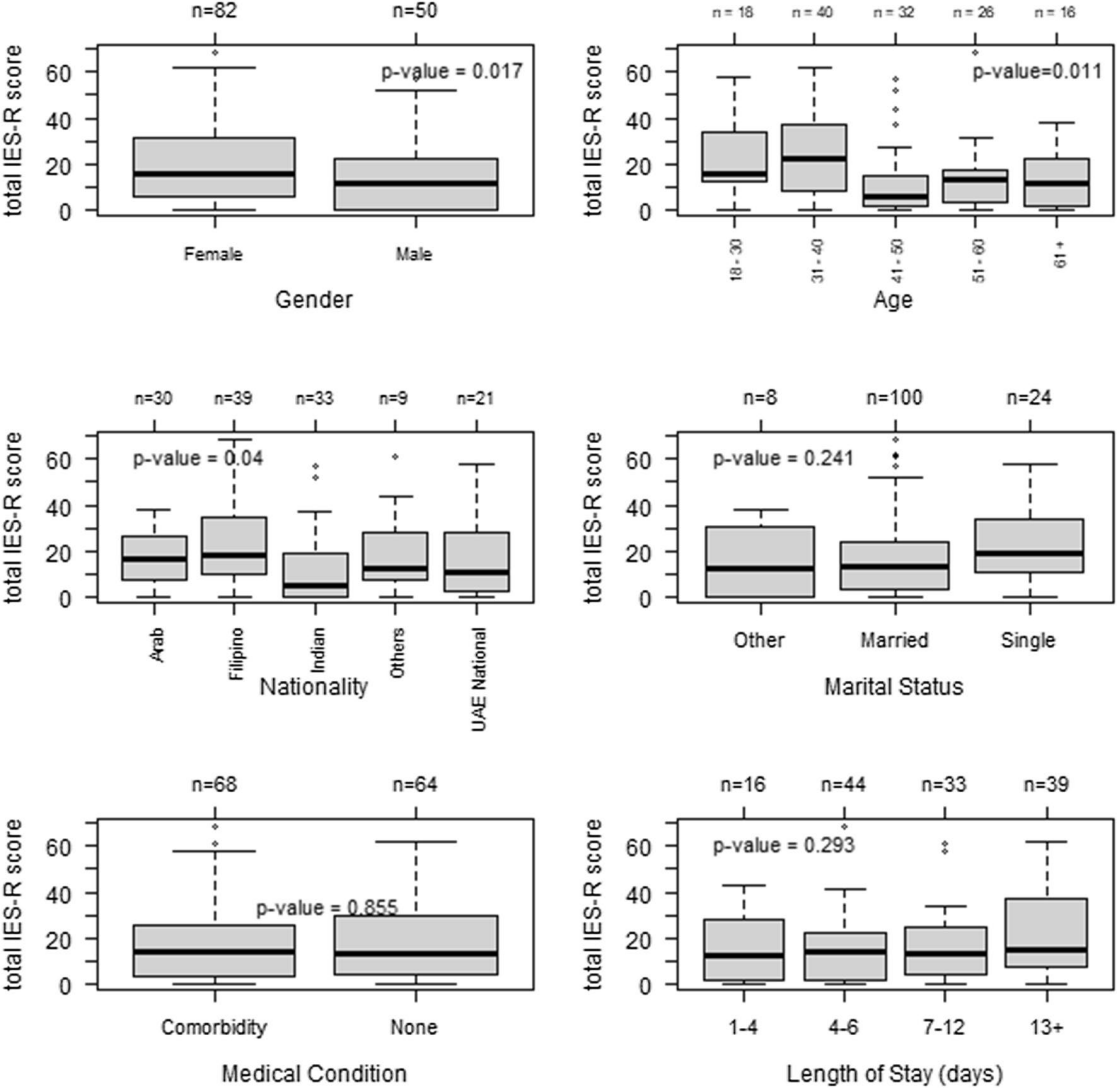
Distribution of the total IES-R score in the gender, age, nationality, and marital status groups. Wilcoxon p-value for gender and Kruskal–Wallis p-value for age, nationality, and marital status.*Other: Divorced/Widow

Figure 1 shows the distribution of the total IES-R score in gender, age, presence of medical condition, nationality, and marital status category. A statistically significant gender difference was found in stress disorder levels (Wilcoxon *p* value = 0.017), with females scoring higher. Furthermore, statistically significant age differences were observed in stress disorder levels (Kruskal Wallis *p* value = 0.011) with those in the 41–50 age group having the lowest levels. Moreover, statistically significant differences in stress disorder levels were observed between the nationality group (*p* = 0.04, Kruskal–Wallis test). No statistically significant difference was observed in medical condition and marital status groups, Kruskal–Willis *p* value of 0.855 and 0.241, respectively.

**Fig. 2:**
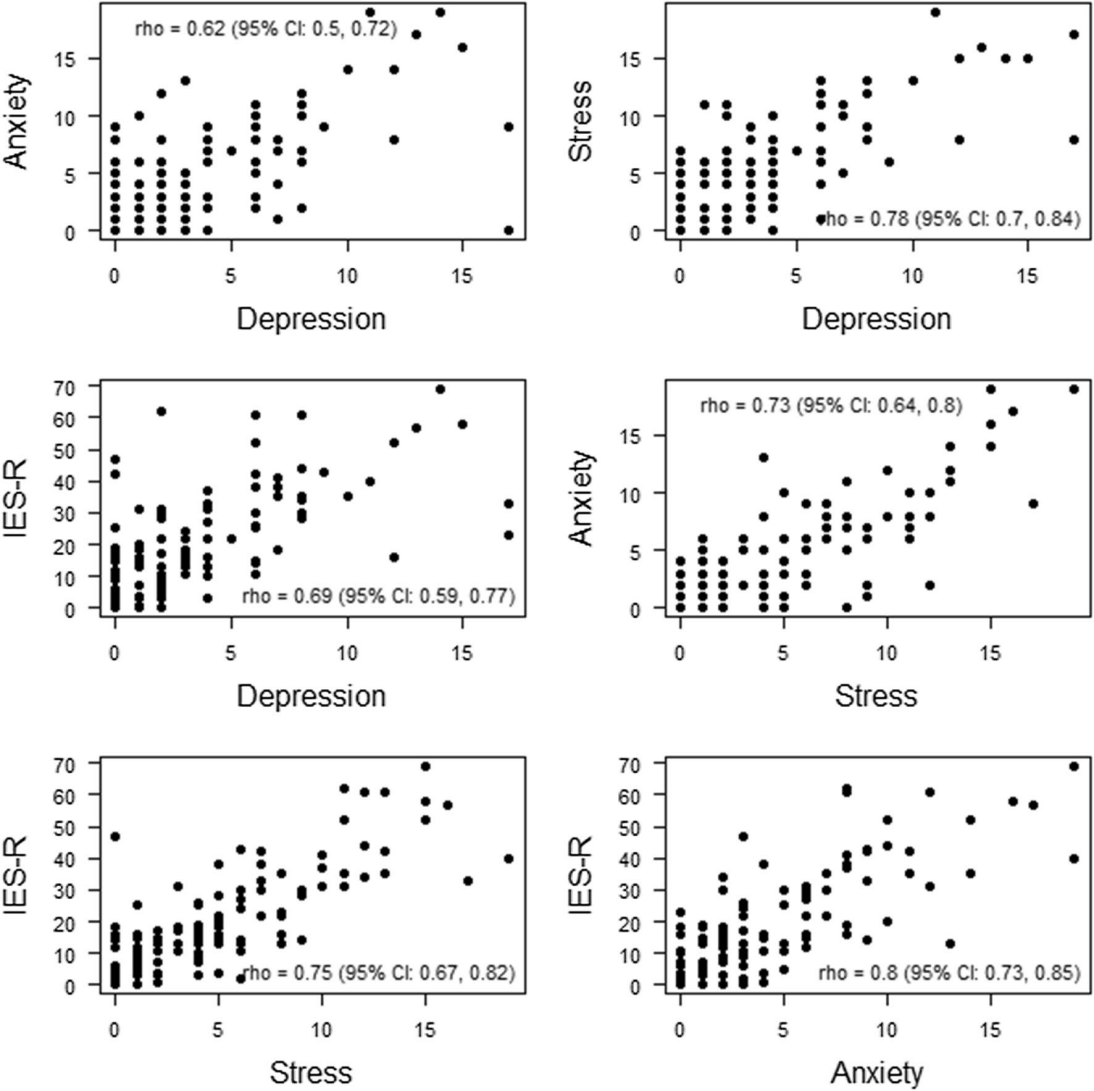
Distribution of IES-R score, Anxiety, Depression, and Stress score, respectively. Spearman rank correlation with corresponding 95% confidence interval. All estimated Spearman correlations were statistically significant, *p* value < 0.001

Figure 2 presents scatterplots of the distribution of IES-R score, Anxiety, Depression, and Stress score, respectively. Spearman rank correlation with corresponding 95% confidence interval was estimated and was all statistically significant *p* value < 0.001.

Table 2 presents the result of the fitted univariate and multivariate logistic (Proportional Odds) ordinal regression model. Univariately, male is less likely to develop depression than female with Odds ratio (OR) = 0.46, and 95% Confidence Interval (CI) = 0.25, 0.87, *p* value = 0.016). However, no statistically significant difference was observed between females and males OR = 0.818 (95% CI 0.389–1.720, *p* value = 0.596) in the multivariate analysis after adjusting for age, presence of medical condition, marital status, nationality and the length of hospital stay. Statistically significant differences were observed between age group (41–50) and (51–60) as compared to the reference group (31–40 years) in univariate and multivariate analysis; OR = 0.233 (95% CI 0.099–0.545, *p* value = 0.001) and 0.352 (95% CI 0.148–0.835, *p* value = 0.018) in univariate analysis, and OR = 0.194 (95% CI 0.077–0.493, *p* value = 0.001) and 0.248 (95% CI 0.089–0.688, *p* value = 0.007) in multivariate analysis, respectively (Table 2). The length of hospital stay was statistically significant in both univariate and multivariate analysis, that is, the risk of stress increases as length of hospital stay increases with an OR = 1.053 (95% CI 1.016–1.091, *p* value = 0.004) for each day of stay in univariate analysis and OR = 1.059 (95% CI 1.010–1.10, *p* value = 0.018) for each day of stay in multivariate analysis. No statistically significant differences were observed for those with a medical condition, however, after adjusting for confounding factors, those with a medical condition had higher levels of psychological distress compared to those without a medical condition; OR = 1.77 (95% CI 0.985–3.489, *p* value = 0.101). Those with Arab nationality were also shown to have higher levels of psychological distress compared to those with Filipino nationality as a reference group, although not statistically significant; OR = 1.72 (95% CI 0.657–4.491, *p* value = 0.270).

**Table 2:** Univariate and multivariate ordinal logistic (proportional odds) regression analysis of factors associated with psychological distress level or an increased score of IES-R score

	*N* (%)	Univariate		Multivariate	
		OR (95% CI)^a^	*Z* (*p* value)^b^	OR (95% CI)^a^	*Z* (*p* value)^b^
Female	82 (62.1%)	Reference		Reference	
Male	50 (37.9%)	0.462 (0.247, 0.865)	– 2.412 (0.016)	0.816 (0.386, 1.705)	– 0.551 (0.582)
Medical condition					
No	64 (48.5%)	Reference		Reference	
Yes	68 (51.5%)	1.058 (0.584, 1.916)	0.186 (0.853)	1.767 (0.985, 3.489)	1.639 (0.101)
Age					
31–40	18 (13.6%)	Ref		Ref	
18–30	40 (30.3%)	0.708 (0.267, 1.877)	– 0.694 (0.488)	1.006 (0.324, 3.122)	0.010 (0.992)
41–50	32 (24.2%)	0.233 (0.099, 0.545)	– 3.359 (0.001)	0.194 (0.077, 0.493)	– 3.448 (0.001)
51–60	26 (19.7%)	0.352 (0.148, 0.835)	– 2.370 (0.018)	0.248 (0.089, 0.688)	– 2.678 (0.007)
61+	16 (12.1%)	0.312 (0.110, 0.886)	– 2.188 (0.029)	0.322 (0.081, 1.280)	– 1.609 (0.108)
Married	8 (6.1%)	Reference		Reference	
Divorced/Widow	100 (75.8%)	0.823 (0.203, 3.340)	– 0.272 (0.786)	0.544 (0.115, 2.581)	– 0.766 (0.444)
Single	24 (18.2%)	1.880 (0.876, 4.034)	1.620 (0.105)	0.580 (0.221, 1.526)	– 1.103 (0.270)
Nationality					
Filipino	30 (22.7%)	Reference		Reference	
Arab	31 (23.5%)	0.637 (0.286, 1.422)	– 1.100 (0.271)	1.718 (0.657, 4.491)	1.103 (0.270)
Indian	9 (6.8%)	0.270 (0.116, 0.627)	– 3.041 (0.002)	0.470 (0.173, 1.280)	– 1.477 (0.140)
Others	39 (29.5%)	0.662 (0.184, 2.385)	– 0.631 (0.528)	0.807 (0.210, 3.102)	– 0.312 (0.755)
UAE	21 (15.9%)	0.379 (0.146, 0.979)	– 2.004 (0.045)	0.587 (0.172, 2.002)	– 0.850 (0.395)
Length of hospital stay median (IQR)	13.5 (4–27.5)	1.053 (1.016, 1.091)	2.860 (0.004)	1.059 (1.010, 1.110)	2.370 (0.018)

Frequency (percentage) for categorical data and median (*IQR* interquartile range) for the length of stay

^a^Estimated odds ratios (OR) with 95% CI

^b^Wald’s *z* value, and corresponding *p* values

## Discussion

To our knowledge, this is the first study to investigate the mental health impact of COVID-19 among COVID-19 inpatients in the UAE. The study results indicated that in the population of hospitalised patients with COVID-19 infection, the proportions of depression, anxiety, and stress levels were low (below screening cut-off) in the majority (90%) of the patients as measured using the DASS-21 scale. Additionally, the IES-R scores were also below the screening cut-off points, which indicates normal levels of psychological distress. Our findings were similar to the findings from other Middle Eastern countries reporting psychological impact scores of the COVID-19 pandemic among their general population as measured using the IES-R scale [14, 15]. Our findings, however, diverge from another UAE study that assessed the impact of COVID-19 on depression and anxiety among healthcare professionals in UAE. The study in question reported 51.5 and 38.3% of study participants experiencing clinically significant levels of anxiety and depressive symptoms, respectively [21]. Unlike the general population in our study, the frontline healthcare professional is known to experience significant work-related stress and anxiety due to work overload as well as fear of COVID-19 infection [22]. A previous study among a similar population to our study has found a high prevalence of psychological distress, namely anxiety and depressive symptoms, among Bangladeshi COVID-19 hospitalised patients [10]. Previous studies have also suggested that incompetent healthcare systems and treatment negligence in the healthcare facilities as possible mechanisms to explain the high prevalence of psychological distress among hospitalised COVID-19 patients [23, 24]. Therefore, we believe the normal levels of psychological distress among our participants in our current study can also be attributed to the health care system in Abu Dhabi and an overall COVID-19 control and management in the UAE.

In this study, we found that the IES-R used in our study was reliable as the Cronbach’s alpha score of the IES-R (Bootstrap 95% confidence interval based on 1000 samples) was 0.93 (95% CI 0.908–0.949). The IES-R scale has been validated and has been frequently used for assessing the psychological impact of COVID-19 infections internationally, for example, in Jordan [15], China [25], Iran [26], and Italy [27]. Our regression analysis revealed that the length of hospital stay was significantly associated with higher levels of psychological distress (depression, anxiety, and stress) as measured using IES-R score, independent of age, gender, medical condition, marital status, and nationality. This finding is in agreement with an integrative review which confirmed that hospitalisation experience negatively affected patients’ psychological well-being and increased feelings of depression and anxiety among adult patients regardless of the reason for admission [28]. An Iranian study on 152 patients with COVID-19 infection also revealed that 26% COVID-19 inpatients reported moderate to high levels of anxiety and depression [26]. Environmental factors (such as sunlight exposure in patients' rooms), social factors (such as financial difficulties), and health-related factors (such as illness severity and disease symptoms) have been shown to contribute to a poor hospitalisation experience [29, 30]. Therefore, we believe the aforementioned mechanisms may also explain our findings with possibly an additional factors from COVID-19-related treatments (such as COVID-19 medication and physical discomfort) [10].

Our study also found that patients aged 41–60 years were significantly associated with lower levels of psychological distress, compared to patients aged 31–40 years. A Chinese study suggested the amount of time spent focusing on COVID-19-related information as the mechanism in explaining more prevalent cases of anxiety and depressive symptoms among those aged 35 years or below, compared to their older counterparts [31]. In addition, another previous study also found that individuals aged 35 years or below were more likely to have anxiety and depressive symptoms during the COVID-19 pandemic [32]. The exact mechanism on the effect of age on the psychological distress among hospitalised patients with COVID-19 is not fully understood.

We did not find any significant associations between other predictors, namely marital status, nationality, or presence of medical condition and psychological distress among hospitalised patients with COVID-19. However, those with medical condition or those with Arab nationality had strong magnitude of associations of psychological distress compared to their respective reference. We have a different finding with an Iranian study that found being divorced was associated with higher psychological load (depression and anxiety) among COVID-19 inpatients [26]. On a different note, the association between presence of medical condition or comorbidity and psychological impact among hospitalised patients has been established. We have a different finding from a previous study in hospitalised patients with COVID-19 found that presence of comorbidity was significantly associated with anxiety and depressive symptoms, independent of COVID-19 severity [10]. Different population characteristics and the limitation of telephone survey to confirm or classify the medical condition in our study may contribute to these differences. Finally, our findings on the association between nationality and psychological impact among COVID-19 inpatients showed that the effect of race on psychological distress due to COVID-19 pandemic especially in the Middle East may be worth further investigation.

## Strengths and Limitations

This is the first study on the psychological impact of COVID-19 among hospitalised COVID-19 patients in the UAE. We were able to control for important confounding factors such as age, gender, presence of medical condition, and length of stay. Our study used two psychological distress scales (IES-R and DASS-21 scales) to assess the levels of anxiety, depression, and stress in our study population. The high intercorrelation between the two scales indicates a high reliability of our data. However, we did not assess the validity of these two scales in this study, thus, studies assessing its validity in this population are warranted.

Despite the aforementioned strengths, our study has several limitations. First, we employed a purposive or subjective sampling design and only included a small number of participants, therefore, not only we had low power to detect differences, but also reduced the study’s external validity or generalizability. However, we recruited the study participants from COVID-19 isolation units in three healthcare facilities in Abu Dhabi, to increase the representativeness of our study samples. We also did not have medical records to compare with their pre-existing psychiatric conditions, and we do not have any control inpatients groups for comparison purposes. Next, we had no follow-up after discharge from hospitals, therefore, the long-term consequences of COVID-19 infection on mental health among our participants are still unknown. The period during which the data was collected between 14 July 2020 and 19 December 2020, may have a bearing on the findings as the changing face of the pandemic at that time period of survey may have affected the responses of participants. The telephone interview methodology may also pose certain challenges as this method only can serve to collect the baseline information on variables of interest, thus, could not include severe COVID-19 inpatients. Lastly, as in other observational studies, this present study is also prone to residual and unmeasured confounding (such as socioeconomic status and medication), which could influence the psychological impact in our population.

## Conclusions

Our results on the psychological impact of COVID-19 among hospitalised patients may help to identify those at risk for psychological distress due to COVID-19 hospitalisation. Early identification and targeted treatment of those at risk for psychological distress due to COVID-19 hospitalisation for targeted prevention and future pandemic preparedness plans. Future studies on the long-term consequences of COVID-19 infection on mental health among ever-hospitalised patients infected with COVID-19 are warranted.

## Data Availability

The datasets used and/or analysed during the current study are available from the corresponding author on reasonable request.

## Abbreviations

COVID-19: Coronavirus disease of 2019
UAE: United Arab Emirates
SARS: Severe acute respiratory syndrome
DASS-21: Depression, Anxiety and Stress Scale—21 Items
RT-PCR: Reverse transcription polymerase chain reaction
IES-R: Impact of Event Scale-Revised
CI: Confidence interval
OR: Odds ratio
